# Hepatorenal Syndrome in the Setting of Intrahepatic Cholangiocarcinoma and Chronic Lymphedema: A Case Report

**DOI:** 10.7759/cureus.67415

**Published:** 2024-08-21

**Authors:** Sean Lief, Mohammed A Khan, Axel B Lichtenberg, Srihita Patibandla, Ali Z Ansari

**Affiliations:** 1 Department of Internal Medicine, William Carey University College of Osteopathic Medicine, Hattiesburg, USA; 2 Department of Internal Medicine, Merit Health Wesley, Hattiesburg, USA; 3 Department of Orthopedic Surgery, William Carey University College of Osteopathic Medicine, Hattiesburg, USA; 4 Department of Internal Medicine, Trinity Health Grand Rapids, Grand Rapids, USA; 5 Department of Pathology and Laboratory Medicine, William Carey University College of Osteopathic Medicine, Hattiesburg, USA

**Keywords:** metastasis, varices, portal hypertension, urinary tract infection, anuria, jaundice, lymphedema, intrahepatic cholangiocarcinoma, acute kidney injury, hepatorenal syndrome

## Abstract

Cholangiocarcinomas are aggressive cancers originating in the bile ducts, classified based on their location as intrahepatic, perihilar, or distal, and often present with symptoms such as jaundice, abdominal pain, and weight loss. Hepatorenal syndrome, a severe complication of liver failure that impairs kidney function and worsens prognosis, further complicates the management of these tumors. We present the case of a 49-year-old Caucasian male who initially sought treatment for jaundice and associated symptoms, including severe itching, gray-colored stools, and fatigue. His medical history, including recent gastric sleeve surgery, obesity, and smoking, along with symptoms of weight loss and increased leg swelling, initially obscured the severity of his condition. Diagnostic imaging and laboratory tests eventually revealed intrahepatic cholangiocarcinoma complicated by acute kidney injury (AKI) and hepatorenal syndrome. Despite the poor prognosis of hepatorenal syndrome, which typically requires liver transplantation for a definitive cure, the patient chose aggressive treatment following stabilization of his renal function. This case highlights the importance of a thorough diagnostic approach in patients presenting with jaundice and vague symptoms, especially as the incidence of cholangiocarcinoma rises, particularly among younger populations. Early and accurate diagnosis, combined with prompt intervention, is crucial for improving patient outcomes in this challenging clinical scenario.

## Introduction

Cholangiocarcinomas are tumors originating from the epithelial lining of bile ducts [1]. Recent literature suggests a rising incidence of these malignancies [1,2]. Globally, the incidence rate of cholangiocarcinoma ranges from 0.3 to six per 100,000 individuals, making it the second most common type of primary liver cancer and accounting for 10-15% of cases. Various risk factors contribute to the development of cholangiocarcinoma, including lifestyle factors (obesity, tobacco use, and alcohol use), infectious agents (hepatitis B virus and *Clonorchis sinensis*), and autoimmune conditions (inflammatory bowel disease and primary sclerosing cholangitis). Many of these risk factors are associated with chronic inflammation within the bile ducts [3,4]. Cholangiocarcinoma is classified anatomically into three categories: perihilar, distal, and intrahepatic [1]. Intrahepatic cholangiocarcinoma is the least common subtype, representing 10-20% of all cholangiocarcinomas [3]. Alongside the rising incidence, mortality rates for cholangiocarcinoma (CCA) are also increasing. For example, in the United States, mortality rates among the African American population rose by 45% between 2004 and 2014 [5].

Hepatorenal syndrome occurs when severe cirrhosis impairs kidney function through vasoconstrictive effects, leading to acute kidney injury and a decreased glomerular filtration rate (GFR), which worsens the prognosis for cirrhotic patients [6,7]. Typically, serum creatinine is used to estimate GFR, but since hepatorenal syndrome is caused by liver failure, it underestimates kidney impairment due to reduced creatinine production by the liver [6]. The only curative treatment for hepatorenal syndrome is a liver transplant; however, even post-transplant, kidney function often remains impaired [7]. The prognosis remains poor, with a three-month mortality rate of approximately 90% [8]. Several risk factors may predict the development of hepatorenal syndrome, including hyponatremia, high plasma renin levels, the degree of hepatomegaly, and the extent of ascites. The literature identifies high-volume paracentesis without adequate albumin repletion and infection as the two most common causes of hepatorenal syndrome. Notably, in cases of hepatorenal syndrome associated with infection, administering albumin alongside the appropriate antibiotic has been shown to reduce mortality [7].

## Case presentation

A 49-year-old Caucasian male presented to the resident clinic this morning for medication refills and evaluation of yellow discoloration of his eyes and skin. The patient reported the onset of severe itching three weeks ago, followed by gray-colored stools one week later. Yesterday, he noticed significant yellowing of his eyes and skin. Additionally, he has experienced fatigue for the past month, along with episodes of diarrhea, gassiness, nausea, and vomiting. He also mentioned that his urine has been brown in color. He took diphenhydramine (Benadryl) for his symptoms, which did not provide relief, though hot showers seem to temporarily alleviate his symptoms.

His medical history includes obesity, for which he underwent gastric sleeve surgery last year without complications, chronic lymphedema, hypertension, obstructive sleep apnea (OSA), and gastroesophageal reflux disease (GERD). Since the surgery, he has lost a total of 140 pounds. He has a history of gallbladder issues from over 20 years ago but did not undergo any surgery for this condition. The patient currently smokes one pack of cigarettes per day and has done so for approximately 37 years. He is a former occasional alcohol user who quit after his bariatric surgery and denies any recreational drug use. The patient's father died at age 60 from cancer that began in his spine and metastasized to his liver.

The patient attributes his nausea and vomiting to the weight-reducing surgery. He has also noticed increased leg swelling over the past two months. The patient denies ever having a colonoscopy or an esophagogastroduodenoscopy (EGD) procedure. The patient denies experiencing abdominal pain, fever, chest pain, shortness of breath, or urinary symptoms. He also reports no recent travel to the coast or other locations, no unusual food consumption, and denies intravenous drug use or current alcohol use. Given the presentation of jaundice, he was admitted for further evaluation of his condition.

**Figure 1:**
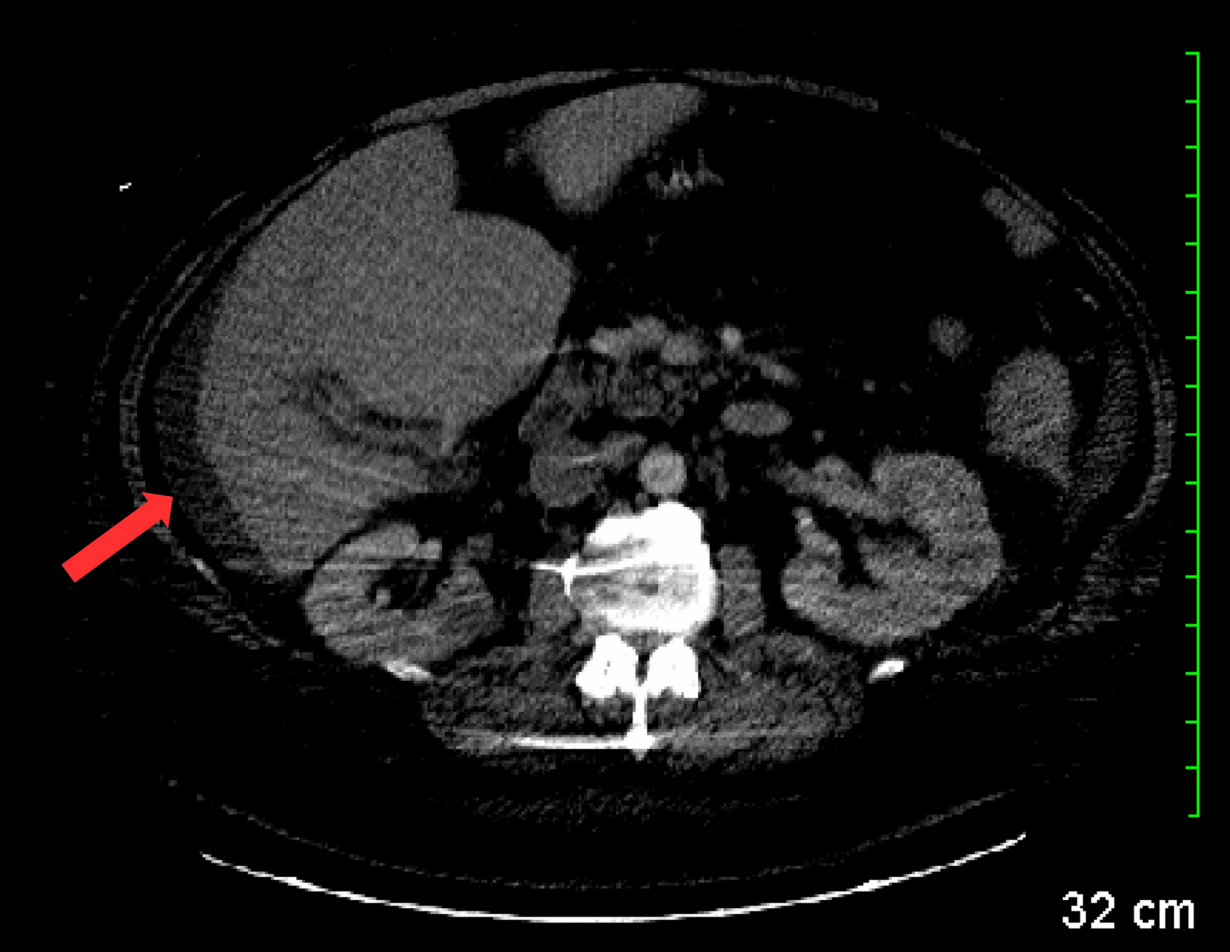
CT scan demonstrating the presence of ascites (red arrow). CT: computed tomography

**Figure 2:**
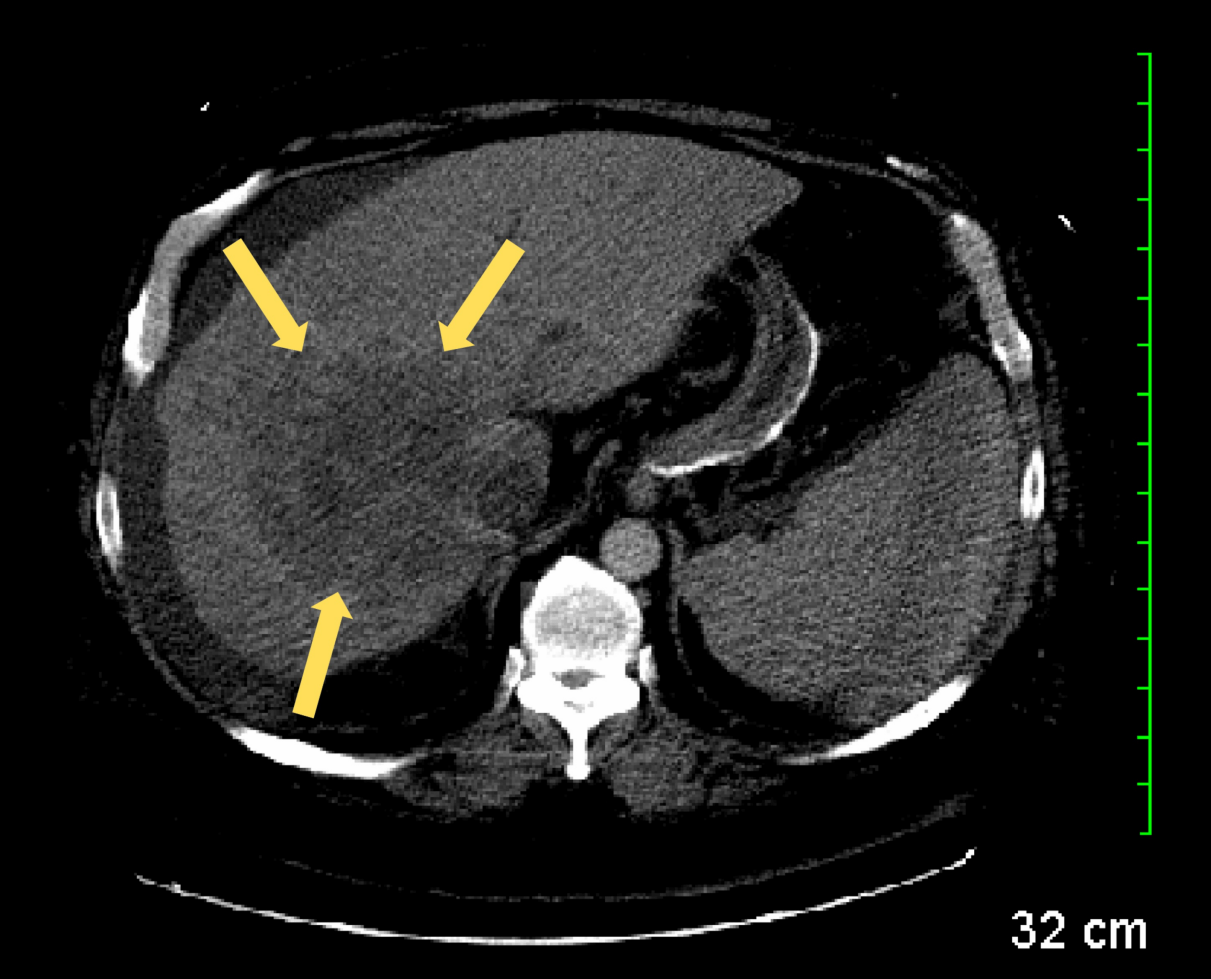
CT scan revealing multifocal masses (yellow arrows) predominantly involving the right lobe of the liver. CT: computed tomography

**Figure 3:**
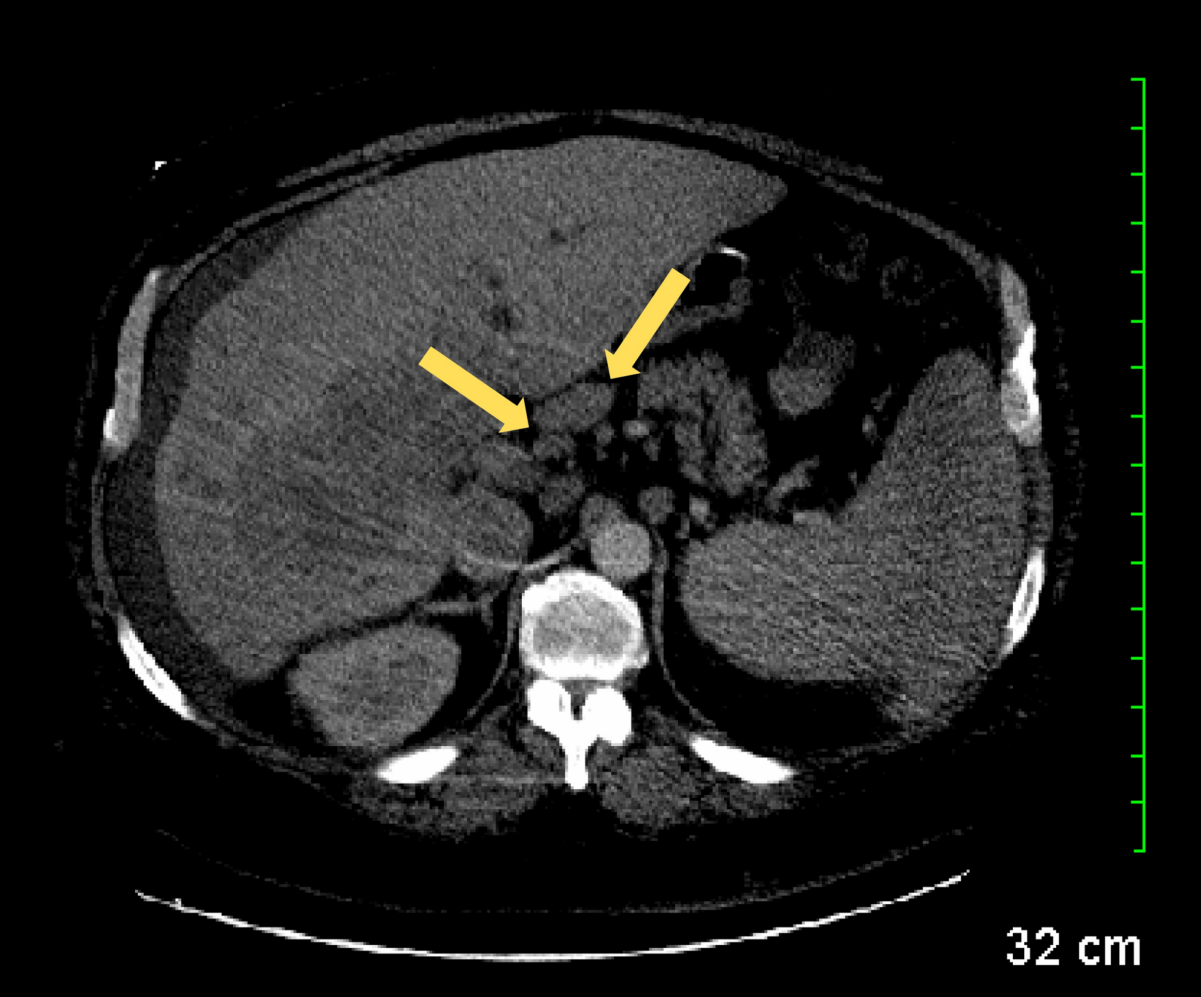
CT scan revealing prominent lymph nodes (yellow arrows) in the porta hepatis region. CT: computed tomography

**Figure 4:**
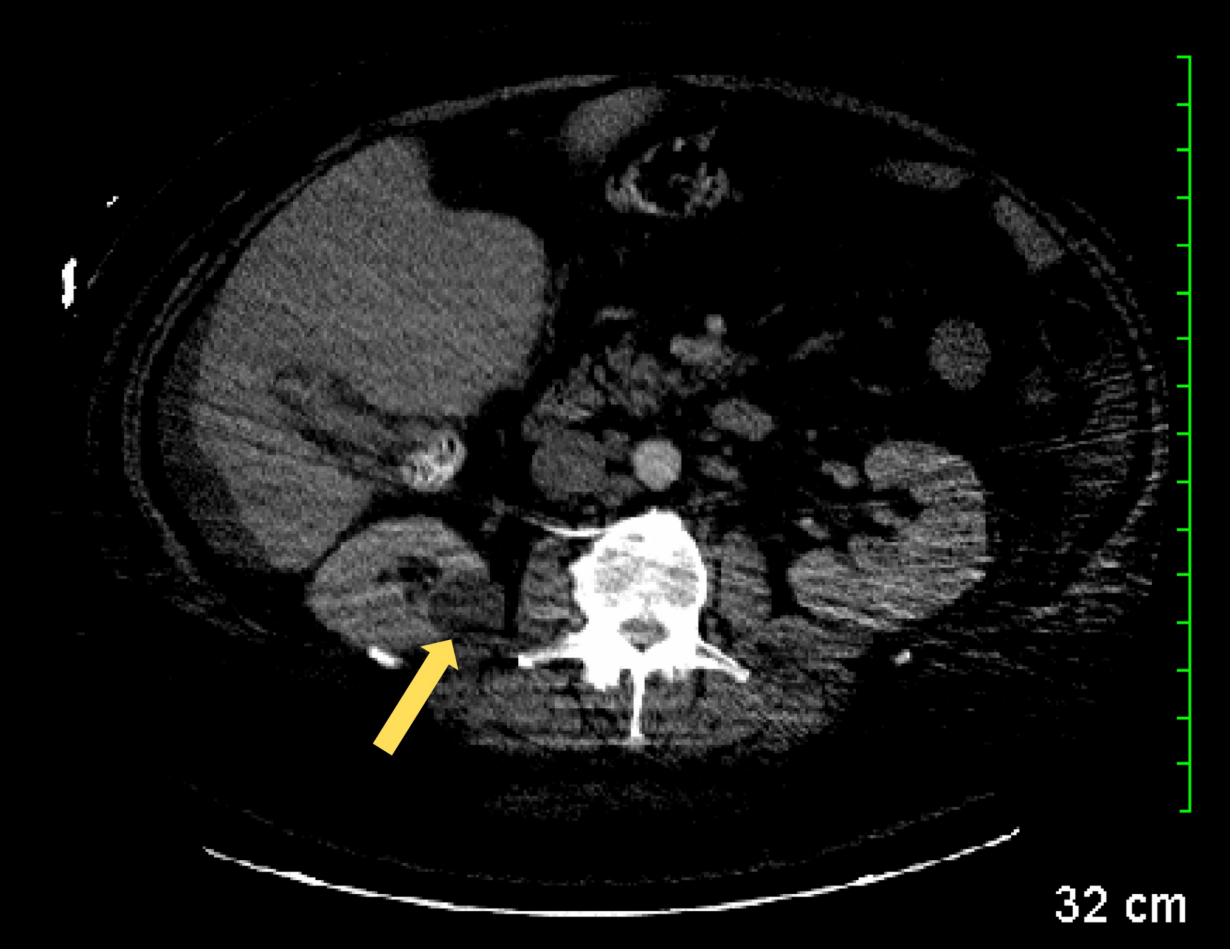
CT scan revealing a right renal cyst (yellow arrow). CT: computed tomography

**Figure 5:**
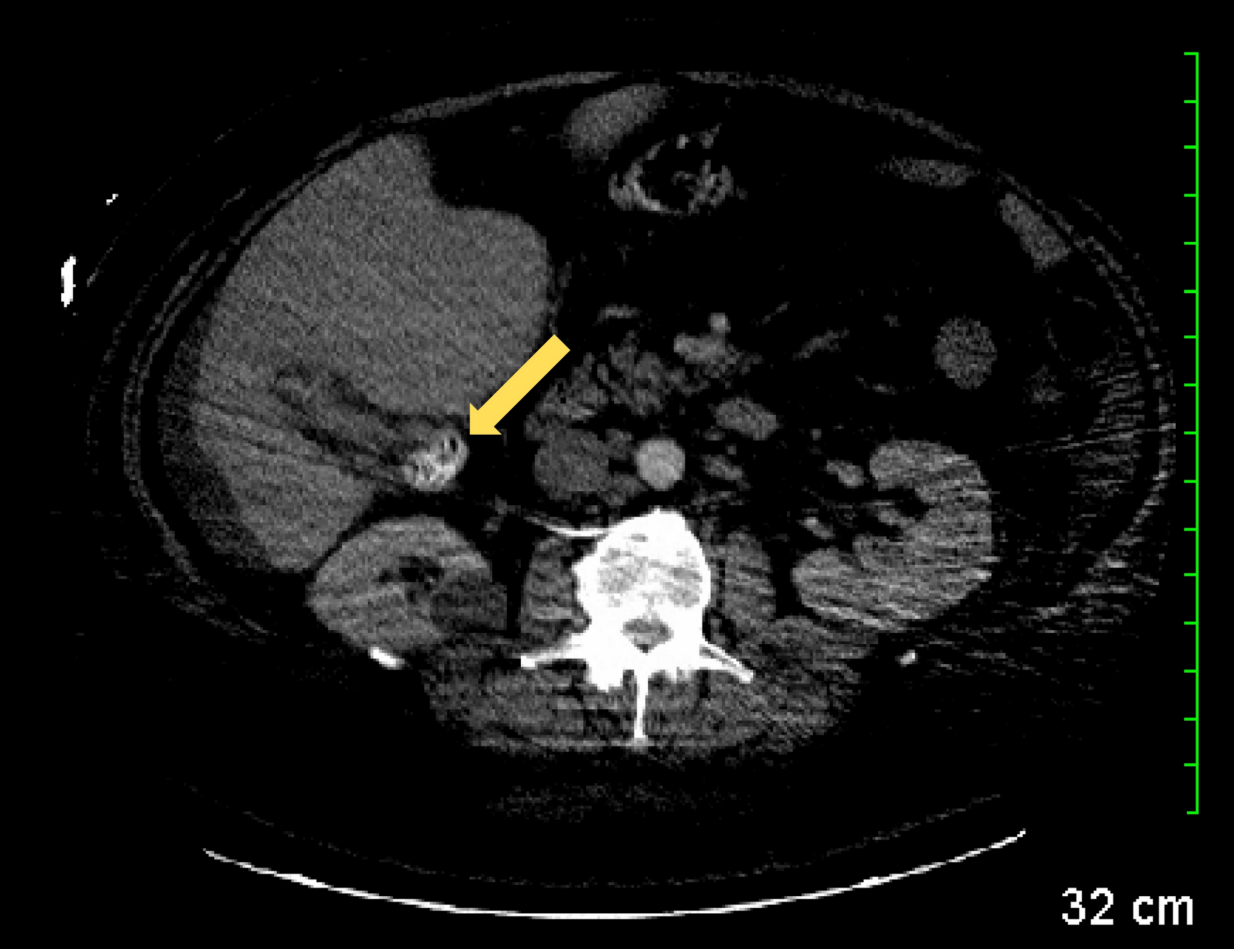
CT scan revealing a partially collapsed gallbladder with small stones inside (yellow arrow). CT: computed tomography

A computed tomography (CT) scan with contrast was performed, revealing a small amount of ascites (Figure 1), multifocal masses involving the right lobe of the liver (Figure 2), prominent nodes in the porta hepatis region (Figure 3), a right renal cyst (Figure 4), and a partially collapsed gallbladder with small stones inside (Figure 5). Laboratory tests showed elevated total bilirubin at 19.3 mg/dL (reference range: 0.1-1.2 mg/dL), aspartate aminotransferase (AST) at 342 U/L (reference range: 10-40 U/L), alanine aminotransferase (ALT) at 42 U/L (reference range: 7-56 U/L), and alkaline phosphatase (ALP) at 282 U/L (reference range: 44-147 U/L), as seen in Table 1.

**Table 1:** Laboratory tests on the day of admission revealing elevated levels of total bilirubin, aspartate aminotransferase, alanine aminotransferase, and alkaline phosphatase.

Test	Observed Value	Reference Range
Total bilirubin	19.3 mg/dL	0.1-1.2 mg/dL
Aspartate aminotransferase	342 U/L	10-40 U/L
Alanine aminotransferase	42 U/L	7-56 U/L
Alkaline phosphatase	282 U/L	44-147 U/L

The next day, the patient underwent an EGD and colonoscopy to search for the primary source of the liver lesions. During the EGD, portal hypertensive gastropathy with varices was identified. New laboratory tests revealed an elevated ALP at 246 U/L (down from 282 U/L), AST at 264 U/L (down from 342 U/L), ALT at 37 U/L (down from 42 U/L), and total bilirubin elevated at 19.5 mg/dL (up from 19.3 mg/dL), as seen in Table 2.

**Table 2:** Laboratory tests on the day following admission revealing elevated levels of total bilirubin, aspartate aminotransferase, alanine aminotransferase, and alkaline phosphatase.

Test	Observed Value	Reference Range
Total bilirubin	19.5 mg/dL	0.1-1.2 mg/dL
Aspartate aminotransferase	264 U/L	10-40 U/L
Alanine aminotransferase	37 U/L	7-56 U/L
Alkaline phosphatase	246 U/L	44-147 U/L

**Figure 6:**
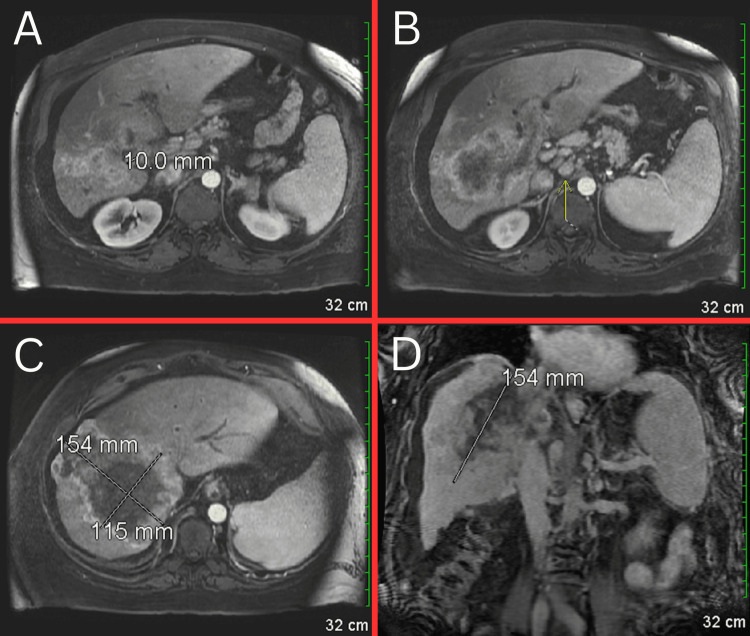
MRI showing ascites around the liver and spleen, as well as trace bilateral pleural effusions. The gallbladder appears markedly abnormal, possibly containing sludge or stones, with some enhancement of the gallbladder wall (Figure 6A). Numerous mildly enlarged nodes (yellow arrow) are noted in the porta hepatis, with the largest measuring 1.5 x 2.0 cm (Figure 6B). A large mass measuring 11.5 x 15 x 15 cm is present, involving a significant portion of the right lobe of the liver (Figure 6C and Figure 6D). The mass extends superiorly through the liver capsule beneath the diaphragm and involves the anterior and posterior segments of the right hepatic lobe, extending into the caudate lobe. MRI: magnetic resonance imaging

A magnetic resonance imaging (MRI) of the abdomen was ordered and revealed a large, heterogeneously enhancing mass occupying a significant portion of the right lobe of the liver (Figure 6). The mass extends through the liver capsule superiorly, just beneath the diaphragm, and involves the porta hepatis, common bile duct, portal vein, and intrahepatic portion of the inferior vena cava. Enlarged nodes in the porta hepatis and para-aortic regions were identified, along with ascites and trace bilateral pleural effusions.

During his hospitalization, the patient complained of itchy skin and was treated with hydroxyzine. A CT-guided liver biopsy was performed, which the patient tolerated well without complications. He was discharged home in stable condition and scheduled an appointment with his primary care doctor to review the results.

Two days later, the patient came to the emergency department (ED) with worsening urinary retention. He reported experiencing increasing pain and a feeling of fullness in his bladder. The symptoms were moderate, and he could not identify anything that made them better or worse. He also reports new-onset abdominal pain, describing it as pressure-like. The pain worsens with movement and is not relieved by any measures.

**Figure 7:**
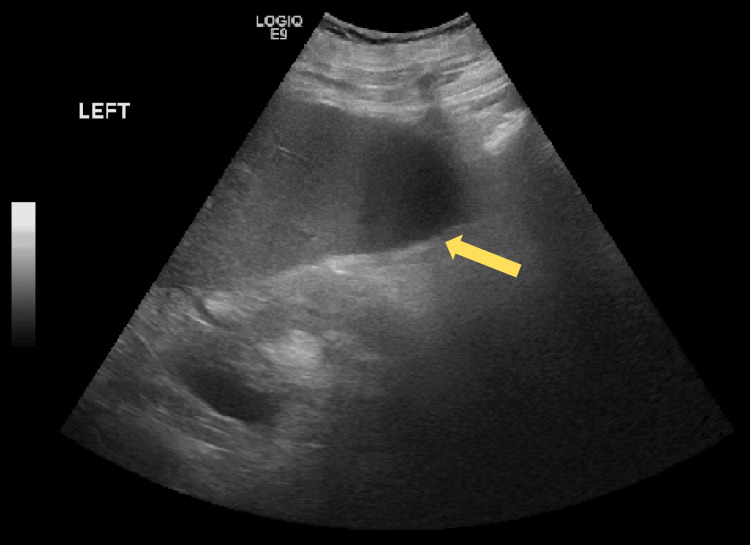
Renal ultrasound revealing ascites (yellow arrow).

A chest X-ray was ordered, which showed no acute cardiopulmonary abnormalities. A urinalysis (UA) revealed a urinary tract infection (UTI), and the patient was treated with 2 grams of intravenous (IV) Rocephin (ceftriaxone). Laboratory results indicated elevated blood urea nitrogen (BUN) and creatinine levels, consistent with acute kidney injury (AKI). Based on these findings and the physical examination, the decision was made to admit the patient to hospitalist services for further evaluation and management. Due to concerns about AKI and anuria, an ultrasound was performed, revealing ascites (Figure 7).

Results from the CT-guided liver biopsy performed during the previous hospitalization revealed adenocarcinoma consistent with cholangiocarcinoma. At the time of initial presentation, the patient was found to have advanced liver disease, evidenced by a prolonged prothrombin time (PT) of 17.1 seconds and an international normalized ratio (INR) of 1.42, along with a decreased albumin level of 2.5 mg/dL, an elevated total bilirubin of 19.3 mg/dL, hyponatremia at 133 mmol/L, and a low creatinine level of 0.55 mg/dL, suggesting acute liver failure without renal impairment at that time. However, during his stay, creatinine levels subsequently rose to a peak of 8.35 mg/dL, representing a more than 15-fold increase from the initial baseline. The patient was not found to be on any nephrotoxic medications, nor was he hypovolemic or in any form of shock. Imaging revealed a benign simple left renal cyst, and further evaluation of urine sediment ruled out intrinsic renal disease. The patient was diagnosed with hepatorenal syndrome in the setting of intrahepatic cholangiocarcinoma. Expressing a strong desire to pursue all available treatment options, the patient chose to aggressively address his diagnosis. Once his renal function stabilized, he continued on dialysis and was subsequently transferred to a comprehensive cancer center for advanced care.

## Discussion

Cholangiocarcinoma is a cancer of the bile ducts characterized by various forms, rising incidence, and poor outcomes [1,2,5]. The three major anatomical subtypes are perihilar, distal, and intrahepatic. Perihilar and distal cholangiocarcinoma are often grouped together and referred to as extrahepatic cholangiocarcinoma [1,9]. The clinical presentation of cholangiocarcinoma is crucial yet highly variable depending on the anatomical subtype. For instance, jaundice is not typically present initially in intrahepatic cholangiocarcinoma, which accounts for 10-15% of cases, but it is the most common symptom of extrahepatic cholangiocarcinoma. Other symptoms are often vague and nonspecific, such as abdominal pain, night sweats, and nausea. Early diagnostic indicators may include elevated liver function tests (LFTs), suggesting liver involvement. Given the numerous conditions that can cause abdominal pain, nausea, and jaundice, clinicians in an acute setting may initially suspect more common causes, such as complications related to gallstones obstructing the common bile duct (choledocholithiasis), rather than a malignant process. Key differentiating factors in clinical presentation include recognizing constitutional symptoms and noting a slower, more insidious onset [9].

Our patient had undergone gastric sleeve surgery 15 months before the diagnosis of intrahepatic cholangiocarcinoma due to morbid obesity. One common complication of this procedure is dumping syndrome, which typically occurs 30 minutes to an hour after meals and is characterized by nausea, abdominal pain, drowsiness, and decreased appetite. Between the surgery and the presentation of jaundice, our patient lost 140 pounds. This significant weight loss, combined with expected procedural complications like dumping syndrome, masked the initial constitutional symptoms of intrahepatic cholangiocarcinoma until the patient presented with the more advanced sign of jaundice [10].

Jaundice initially presents in the sclera at lower serum bilirubin levels and becomes apparent in the skin when levels exceed 2.4 mg/dL [10]. Upon detection or suspicion of jaundice, it is crucial to determine whether it is due to conjugated bilirubin, unconjugated bilirubin, or an unrelated pathogenesis, such as Addison's disease. This distinction can be made through laboratory tests and a thorough history and physical examination. Diagnostic tests to consider include a complete blood count (CBC), alkaline phosphatase (ALP), LFTs, PT/INR, albumin, and most importantly, fractionated bilirubin. Elevated levels of unconjugated and total bilirubin suggest hemolysis, warranting an investigation for potential causes. Conversely, if both total and conjugated bilirubin levels are elevated, ultrasonography should be performed to detect possible strictures or gallstones, and the patient should be evaluated for hepatitis. If no diagnosis is found through initial testing, further investigation should include antibody testing for primary sclerosing cholangitis or primary biliary cholangitis. Additional diagnostic imaging modalities, such as endoscopic retrograde cholangiopancreatography (ERCP), contrast-enhanced CT, and contrast-enhanced MRI, should also be considered [10,11].

Cholangiocarcinoma-induced liver failure leading to hepatorenal syndrome is a rare and not well-documented phenomenon. Hepatorenal syndrome is one of the most severe complications of liver failure, with a particularly poor prognosis [12]. It is thought to result from widespread circulatory dysfunction due to cirrhosis or acute liver failure. Cirrhosis decreases systemic vascular resistance due to portal hypertension causing splanchnic arterial vasodilation. In the early stages, cardiac output compensates for this reduced resistance, but in advanced liver failure, cardiac output can no longer keep up. As a result, the renin-angiotensin-aldosterone system is activated to maintain arterial pressure, leading to renal vasoconstriction, decreased serum sodium, and reduced GFR. This compromises kidney perfusion and results in renal damage. Treating hepatorenal syndrome requires addressing both the compromised kidney perfusion and the underlying liver failure. Vasoactive drugs such as norepinephrine or terlipressin, combined with albumin, can improve survival and reduce kidney injury. However, the only curative treatment for hepatorenal syndrome remains liver transplantation [6-8,12].

The combination of cholangiocarcinoma and hepatorenal syndrome presents a challenging clinical scenario, particularly with intrahepatic cholangiocarcinoma, as liver transplantation has historically been contraindicated due to poor outcomes. However, recent studies have shown promising results for patients with unresectable intrahepatic cholangiocarcinoma when treated with adjuvant chemotherapy, with some studies reporting one-year survival rates as high as 100% [13,14]. Despite these positive findings, liver transplantation remains a controversial treatment option. For patients with systemic disease where surgical resection or transplantation is not feasible, the first-line chemotherapy regimen includes gemcitabine and cisplatin. Emerging targeted therapies, particularly immune checkpoint inhibitors like programmed death-1 (PD-1) and cytotoxic T-lymphocyte antigen-4 (CTLA-4) inhibitors, are showing promising results in the literature. Notably, the antibody treatment durvalumab, when combined with the traditional chemotherapy regimen, has demonstrated a 20% reduction in the risk of death, leading to proposals for its inclusion in the first-line treatment regimen [14]. The potential benefit of liver transplantation in cases of intrahepatic cholangiocarcinoma-induced hepatorenal syndrome remains uncertain, but further research could provide valuable insights into this unique clinical situation.

In light of the rising global incidence of cholangiocarcinoma, it is important to recognize the known risk factors associated with its development [1]. Gender plays a significant role, with men having a higher incidence of cholangiocarcinoma compared to women. Additionally, the incidence is notably higher in Southeast Asian countries like Thailand, which is often attributed to parasitic infections such as *Clonorchis sinensis* or gallstones obstructing the liver's biliary ducts. Chronic inflammation and bile stasis are key factors in cholangiocarcinoma pathogenesis, and some studies have shown a correlation between obesity and the development of intrahepatic cholangiocarcinoma. Our patient had a history of unspecified gallbladder issues for the past 20 years and obesity, which may have contributed to the development of this malignancy. Notably, alcohol use alone has not been identified as a significant risk factor for cholangiocarcinoma [1]. Recent research indicates a steady rise in the incidence of intrahepatic cholangiocarcinoma among younger female cohorts. This trend is not unique to intrahepatic cholangiocarcinoma, as many other malignancies are increasingly being diagnosed in younger populations. Future research must focus on identifying potential carcinogens or previously unrecognized risk factors to better understand and address this shift [15].

This case provides valuable insights into the unexpected presentation and manifestation of intrahepatic cholangiocarcinoma leading to acute kidney injury in the context of acute liver failure. It highlights potential complications clinicians may anticipate and address. When a patient presents with jaundice, we recommend a stepwise diagnostic approach, including appropriate lab work and imaging modalities [10,11]. Given our patient's history of gastric sleeve surgery, a comprehensive laboratory evaluation should include LFTs as well as biomarkers of hepatic synthetic function, such as PT/INR, albumin, serum sodium, creatinine, and bilirubin. For imaging, an initial contrast-enhanced CT scan is recommended to identify underlying pathology, followed by a more targeted modality, such as ERCP, as indicated. Diagnosticians should be aware of the symptoms associated with such malignancies and recognize that patients may not always realize they are acutely ill, especially when symptoms mimic expected procedural complications and desired outcomes [9,10]. In cases of hepatorenal syndrome, it is crucial for clinicians to identify its features, understand its prognostic implications, and be familiar with available treatment options [6-8,12]. Despite the poor prognosis associated with cholangiocarcinoma, current treatment options include surgical resection, chemotherapy, immunotherapy, and liver transplantation [13,14]. In patients initially presenting with locally unresectable cholangiocarcinoma who were treated with chemotherapy and radiotherapy until resection became feasible, one study reported a one-year survival rate of 80.8% and a five-year survival rate of 23.6% [16]. As the global incidence and age of onset for cholangiocarcinoma increase, it is essential for medical providers to maintain a high index of suspicion to ensure timely and accurate diagnosis and treatment [1,15].

## Conclusions

This case highlights the complexity of managing intrahepatic cholangiocarcinoma, particularly when complicated by hepatorenal syndrome. The patient’s initial symptoms were obscured by weight loss and complications from surgery. Over time, these issues developed into severe liver failure, which was further complicated by AKI. The combination of advanced liver disease and renal impairment poses significant treatment challenges, with liver transplantation being the only definitive cure, though often contraindicated in advanced cases. This emphasizes the importance of thorough diagnostic evaluation for patients who present with jaundice and other vague symptoms. With the incidence of cholangiocarcinoma on the rise, particularly among younger populations, heightened awareness, and prompt intervention are crucial for improving patient prognosis.
